# Optimal bounds for Neuman-Sándor mean in terms of the convex combination of the logarithmic and the second Seiffert means

**DOI:** 10.1186/s13660-017-1516-7

**Published:** 2017-10-10

**Authors:** Jing-Jing Chen, Jian-Jun Lei, Bo-Yong Long

**Affiliations:** School of Mathematical Science, Anhui University, Hefei, 230601 China

**Keywords:** 26E60, Neuman-Sándor mean, logarithmic mean, the second Seiffert mean

## Abstract

In the article, we prove that the double inequality

$$ \alpha L(a,b)+(1-\alpha)T(a,b)< \mathit{NS}(a,b)< \beta L(a,b)+(1-\beta)T(a,b) $$

holds for $a,b>0$ with $a\ne b $ if and only if $\alpha\ge1/4$ and $\beta\le1-\pi/[4\log(1+\sqrt{2})]$, where $\mathit{NS}(a,b)$, $L(a,b)$ and $T(a,b)$ denote the Neuman-Sándor, logarithmic and second Seiffert means of two positive numbers *a* and *b*, respectively.

## Introduction

For $a,b>0$ with $a\neq b$, the Neuman-Sándor mean $\mathit {NS}(a,b)$ [1], the second Seiffert mean $T(a,b)$ [2], and the logarithmic mean $L(a,b)$ [1] are defined by

1.1 $$\begin{aligned}& \mathit {NS}(a,b)=\frac{a-b}{2\sinh^{-1}[(a-b)/(a+b)]}, \end{aligned}$$

1.2 $$\begin{aligned}& T(a,b)=\frac{a-b}{2\tan^{-1}[(a-b)/(a+b)]}, \\& L(a,b)=\frac{a-b}{\log a-\log b}, \end{aligned}$$

respectively. It can be observed that the logarithmic mean $L(a,b)$ can be rewritten as (see as [1])

1.3 $$\begin{aligned}& L(a,b)=\frac{a-b}{2\tanh^{-1}[(a-b)/(a+b)]}, \end{aligned}$$

where $\sinh^{-1}(x)=\log(x+\sqrt{1+x^{2}})$, $\tanh^{-1}(x)= \log{\sqrt{(1+x)/(1-x)}}$ and $\tan^{-1}(x)=\arctan(x)$, are the inverse hyperbolic sine, inverse hyperbolic tangent, and inverse tangent, respectively.

Recently, the means *NS*, *T*, *L* and other means have been the subject of extensive research. In particular, many remarkable inequalities for the Neuman-Sándor, second Seiffert and logarithmic means can be found in the literature [2–16].

Let $P(a,b)=(a-b)/(2\sin^{-1}[(a-b)/(a+b)])$, $S(a,b) = \sqrt{( {a^{2}} + {b^{2}})/2}$, $A(a,b)=(a+b)/2$, $I(a,b) = 1/e{({b^{b}}/ {a^{a}})^{1/(b - a)}}$, $G(a,b)=\sqrt{ab}$, and $H(a,b)=2ab/(a+b)$ denote the first Seiffert, root-square, arithmetic, identric, geometric, and the harmonic means of two positive numbers *a* and *b* with $a\ne b$, respectively. Then it is well known that the inequality

$$ S(a,b)>T(a,b)>\mathit {NS}(a,b)>A(a,b)>I(a,b)>P(a,b)>L(a,b)>G(a,b)>H(a,b) $$

holds for $a,b>0$ with $a\ne b$.

In [17] and [18], the authors proved that the double inequalities

$$\begin{aligned}& S(a,b)^{\alpha_{3}}A^{1-\alpha_{3}}(a,b)< \mathit {NS}(a,b)< S(a,b)^{\beta_{3}}A ^{1-\beta_{3}}(a,b), \\& \alpha_{4}S(a,b)+(1-\alpha_{4})G(a,b)< \mathit {NS}(a,b)< \beta_{4}S(a,b)+(1-\beta _{4})G(a,b) \end{aligned}$$

hold for all $a,b>0$ with $a\neq b$ if and only if $\alpha_{3}\leq1/3$, $2(\log(2+\sqrt{2})-\log3)/\log2 \leq\beta_{3}\leq1$, $\alpha_{4}\leq2/3$ and $\beta_{4}\geq1/[\sqrt{2}\log(1+ \sqrt{2})]$.

In [19], it was showed that the inequality

$$ {P^{\alpha_{2}}}(a,b){T^{1 - \alpha_{2}}}(a,b) < \mathit {NS}(a,b) < {P^{\beta _{2}}}(a,b){T^{1 - \beta_{2}}}(a,b) $$

holds for all $a,b>0$ with $a\ne b $ if and only if $\alpha_{2}>1/3$ and

$$ \beta_{2} \leq\log\biggl(\frac{4\log(1+\sqrt{2})}{\pi}\biggr)/\log2 =0.1663 \ldots. $$

Let $L_{p}(a,b)=(a^{p+1}+b^{p+1})/(a^{p}+b^{p})$ be the Lehmer mean of two positive numbers *a* and *b* with $a\neq b$. In [10], the authors proved the double inequality

$$ L_{\alpha_{1}}(a,b)< \mathit {NS}(a,b)< L_{\beta_{1}}(a,b) $$

holds for all $a,b>0$ with $a\neq b$ if and only if $\alpha_{1} =1.8435 \ldots$ is the unique solution of the equation $(p+1)^{1/p}=2\log(1+ \sqrt{2})$, and $\beta_{1} =2$.

Let

$$ M_{p}(a,b)= \textstyle\begin{cases} (\frac{a^{p}+b^{p}}{2})^{1/p}, & p\neq0, \\ \sqrt{ab}, & p=0, \end{cases} $$

be the *p*th power means of two positive numbers *a* and *b* with $a\neq b$. In [20], the authors proved the sharp double inequality

$$ {M_{\log2/(\log\pi- \log2)}}(a,b) < T(a,b) < {M_{5/3}}(a,b) $$

holds.

Gao [21] proved the optimal double inequality

$$ I(a,b) < T(a,b) < \frac{{2e}}{\pi}I(a,b) $$

holds for all $a,b>0$ with $a\ne b$.

Yang [22] proved the inequality

$$ A_{p}^{1/(3p)}(a,b){G^{1 - 1/(3p)}}(a,b) < L(a,b) < A_{q}^{1/(3q)}(a,b) {G^{1 - 1/(3q)}}(a,b) $$

holds for all $a,b>0$ with $a\ne b$ if and only if $p\geq1/ \sqrt{5}$ and $0< q\leq1/3$. And the inequality

$$ M_{0}(a,b)< L(a,b)< M_{1/3}(a,b) $$

was proved by Lin in [23].

In [24], the authors present bounds for *L* in terms of *G* and *A*

$$ G^{2/3}(a,b)A^{1/3}(a,b)< L(a,b)< \frac{2}{3}G(a,b)+ \frac{1}{3}A(a,b) $$

for all $a,b>0$ with $a\neq b$.

The purpose of this paper is to answer the following questions: What are the least value *α* and the greatest value *β* such that

$$ \alpha L(a,b) + (1 - \alpha)T(a,b) < \mathit {NS}(a,b) < \beta L(a,b) + (1 - \beta)T(a,b) $$

holds for all $a,b>0$ with $a \ne b$ ?

## Lemmas

It is well known that, for $x \in(0,1) $,

2.1 $$\begin{aligned}& \tanh^{-1} (x) =x+\frac{x^{3}}{3}+\frac{x^{5}}{5}+\cdots=\sum ^{ \infty}_{n=0}\frac{1}{2n+1}x^{2n+1}, \end{aligned}$$

2.2 $$\begin{aligned}& \tan^{-1}(x)=x-\frac{x^{3}}{3}+\frac{x^{5}}{5}- \frac{x^{7}}{7}+\cdots =\sum^{\infty}_{n=0} \frac{(-1)^{n}}{2n+1}x^{2n+1}. \end{aligned}$$

To establish our main result, we need several lemmas as follows.

### Lemma 2.1

[25]

*Let*

$$\begin{aligned}& H(x) = \frac{1}{\sinh^{-1}x}- \frac{x}{\sqrt{1+x^{2}}(\sinh^{-1}x)^{2}}. \end{aligned}$$

*Then* $H(x)$ *is strictly increasing on* $(0,1)$. *Moreover*, *the inequality*

2.3 $$\begin{aligned}& H(x)< \frac{x}{3}-\frac{x^{3}}{9} \end{aligned}$$

*holds for any* $x \in(0,0.76) $ *and the inequality*

2.4 $$\begin{aligned}& H(x)>\frac{x}{3}-\frac{17x^{3}}{90} \end{aligned}$$

*holds for any* $x \in(0,1) $.

### Lemma 2.2

*Let* $S(x) = 1/{\tanh^{ - 1}}x - x/[(1 - {x^{2}}){({\tanh^{ - 1}}x)^{2}}] $. *Then*

2.5 $$\begin{aligned}& S(x)< -\frac{2}{3}x-\frac{1}{3}x^{3}- \frac{1}{3}x^{5} \end{aligned}$$

*for any* $x \in(0,1) $ *and*

2.6 $$\begin{aligned}& S(x)>-\frac{2}{3}x-x^{3}-\frac{x^{5}}{4} \end{aligned}$$

*for any* $x \in(0,0.76)$.

### Proof

Let

$$\begin{aligned}& G(x)=\bigl(1-x^{2}\bigr) \bigl(\tanh^{-1}x \bigr)^{2}\biggl[S(x)+\frac{2}{3}x+\frac{1}{3}x^{3}+ \frac{1}{3}x^{5}\biggr]. \end{aligned}$$

Then direct computation leads to

2.7 $$\begin{aligned}& G(0)=0, \end{aligned}$$

2.8 $$\begin{aligned}& G'(x)=\frac{1}{3}g(x)\tanh^{-1}x, \end{aligned}$$

where $g(x) = (2 - 7{x^{6}} - 3{x^{2}}){\tanh^{ - 1}}x + 2{x^{5}} + 2 {x^{3}} - 2x$. It follows that

2.9 $$\begin{aligned}& g'(x)=\frac{1}{1-x^{2}}g_{1}(x), \end{aligned}$$

where ${g_{1}}(x) = ( - 42{x^{5}} - 6x)(1 - {x^{2}}){\tanh^{ - 1}}x - 17{x^{6}} + 4{x^{4}} + 5{x^{2}}$. Considering (2.1), we have

2.10 $$\begin{aligned} {g_{1}}(x)& < \bigl( - 42{x^{5}} - 6x\bigr) \bigl(1 - {x^{2}}\bigr) \biggl( x + \frac{x^{3}}{3} + \frac{x^{5}}{5} \biggr) - 17{x^{6}} + 4{x^{4}} + 5{x^{2}} \\ & = \frac{1}{5}\bigl(42{x^{12}} + 28{x^{10}} + 146{x^{8}} - 291{x^{6}} + 40 {x^{4}} - 5{x^{2}}\bigr) \\ & < x^{2}\bigl(216x^{6}-291x^{4}+40x^{2}-5 \bigr)< 0, \end{aligned}$$

for $x \in(0,1) $. Thus, (2.9) and (2.10) as well as $g(0)=0$ imply $g(x)<0$ for $x \in(0,1) $. Therefore, combining (2.7) and (2.8), we get $G(x)<0$ for $x \in(0,1) $. It means inequality (2.5) holds.

Let

$$ Q(x)=\bigl(1-x^{2}\bigr) \bigl(\tanh^{-1}x \bigr)^{2}\biggl[S(x)+\frac{2}{3}x+x^{3}+ \frac{x^{5}}{4}\biggr]. $$

Direct computation leads to

2.11 $$\begin{aligned}& Q(0)=0, \end{aligned}$$

2.12 $$\begin{aligned}& Q'(x)=\frac{1}{12}q_{1}(x)\tanh^{-1}x, \end{aligned}$$

where

$$ q_{1}(x)=6x^{5}+24x^{3}-8x+ \bigl(8+12x^{2}-45x^{4}-21x^{6}\bigr) \tanh^{-1}x. $$

When $x\in(0,0.7]$, considering (2.1) and the fact $8+12x^{2}-45x ^{4}-21x^{6}=(3-21x^{6})+(5+12x^{2}-45x^{4})>0 $, we can get

$$\begin{aligned} q_{1}(x)&>6x^{5}+24x^{3}-8x+ \bigl(8+12x^{2}-45x^{4}-21x^{6}\bigr) \biggl(x+ \frac{x ^{3}}{3}+\frac{x^{5}}{5}\biggr) \\ & =-\frac{21}{5}x^{11}-16x^{9}- \frac{168}{5}x^{7}-\frac{167}{5}x^{5}+ \frac{116}{3}x^{3} \\ & >x^{3}\biggl(-\frac{269}{5}x^{4}- \frac{167}{5}x^{2}+\frac{116}{3}\biggr)>0. \end{aligned}$$

When $x\in(0.7,0.76)$, direct computation leads to

2.13 $$\begin{aligned}& q_{1}(0.76)=1.8639\ldots>0, \end{aligned}$$

2.14 $$\begin{aligned}& q_{1}'(x)=q_{2}(x)/\bigl(1-x^{2} \bigr), \end{aligned}$$

where $q_{2}(x)=92x^{2}-87x^{4}-51x^{6}+(126x^{7}+54x^{5}-204x^{3}+24x) \tanh^{-1}x$. Considering (2.1) and the fact $126x^{7}+54x^{5}-204x ^{3}+24x<12x(15x^{4}-17x^{2}+2)<0$, we can get

2.15 $$\begin{aligned} q_{2}(x)&< 92x^{2}-87x^{4}-51x^{6}+ \bigl(126x^{7}+54x^{5}-204x^{3}+24x\bigr) \biggl(x+\frac{x ^{3}}{3}+\frac{x^{5}}{5}\biggr) \\ & =\frac{126}{5}x^{12}+\frac{264}{5}x^{10}+ \frac{516}{5}x^{8}- \frac{301}{5}x^{6}-283x^{4}+116x^{2} \\ & < 2x^{4}\bigl(91x^{4}-30x^{2}-20 \bigr)+x^{2}\bigl(116-243x^{2}\bigr)< 0. \end{aligned}$$

Thus, (2.13)-(2.15) imply that

2.16 $$\begin{aligned}& q_{1}(x)>0 \end{aligned}$$

holds for any $x\in(0.7,0.76)$.

Therefore, $Q(x)>0$ for $x\in(0, 0.76)$ follows from (2.11), (2.12) and (2.16). That means inequality (2.6) holds. □

### Lemma 2.3

*Let* $T(x) = 1/{\tan^{ - 1}}x - x/[(1 + {x ^{2}}){({\tan^{ - 1}}x)^{2}}] $. *Then*

2.17 $$\begin{aligned}& T(x)< \frac{2}{3}x-\frac{1}{3}x^{3}+ \frac{2}{7}x^{5} \end{aligned}$$

*for any* $x\in(0,1)$ *and*

2.18 $$\begin{aligned}& T(x)>\frac{2}{3}x-\frac{2}{5}x^{3}+\frac{x^{5}}{7} \end{aligned}$$

*for any* $x\in(0,0.76)$.

### Proof

Let

$$\begin{aligned}& M(x)=\biggl[T(x)-\frac{2}{3}x+\frac{x^{3}}{3}-\frac{2}{7}x^{5} \biggr]\bigl(1+x^{2}\bigr) \bigl( \tan^{-1}x \bigr)^{2}. \end{aligned}$$

Differentiating $M(x)$, we have $M'(x) = [t(x){\tan^{ - 1}}x]/21 $, where

$$ t(x) = 14x + 14{x^{3}} - 12{x^{5}} + \bigl( - 42{x^{6}}+5{x^{4}} - 21{x ^{2}} - 14\bigr){ \tan^{ - 1}}x. $$

For $x \in(0,1) $, we have $- 42{x^{6}}+5{x^{4}} - 21{x^{2}} - 14 <-42x ^{6}-16x^{2}-14< 0 $. Thus from (2.2), we can get

$$\begin{aligned} t(x) &< 14x+14x^{3}-12x^{5}+\bigl(- 42{x^{6}}+5{x^{4}} - 21{x^{2}} - 14\bigr) \biggl(x-\frac{x ^{3}}{3}\biggr) \\ & = 14{x^{9}} - \frac{131}{3}x^{7} - \frac{7}{3}x^{3} \\ & < - \frac{89}{3}x^{7} -\frac{7}{3}x^{3} < 0. \end{aligned}$$

Therefore $M'(x)<0 $ for $x\in(0,1) $. Considering the fact $M(0)=0$, we get $M(x)<0$ for $x\in(0,1)$. So the inequality (2.17) holds.

Let

$$\begin{aligned}& N(x)=\biggl[T(x)-\frac{2}{3}x+\frac{2}{5}x^{3}- \frac{x^{5}}{7}\biggr]\bigl(1+x^{2}\bigr) \bigl( \tan^{-1}x \bigr)^{2}. \end{aligned}$$

Differentiating $N(x)$, we have $N'(x) = n(x){\tan^{ - 1}}x $, where

$$ n(x) = \frac{2}{3}x + \frac{4}{5}x^{3}- \frac{2}{7}x^{5}-\biggl(x^{6}- \frac{9}{7}x^{4}+ \frac{4}{5}x^{2}+\frac{2}{3}\biggr)\tan^{-1}x. $$

Because of

$$ \biggl(\frac{4}{5}x^{2}-\frac{9}{7}x^{4} \biggr)+x^{6}+\frac{2}{3}>0 $$

for $x\in(0,0.76)$, it follows that

$$\begin{aligned} n(x)&>\frac{2}{3}x + \frac{4}{5}x^{3}- \frac{2}{7}x^{5}-\biggl(x^{6}- \frac{9}{7}x^{4}+ \frac{4}{5}x^{2}+\frac{2}{3}\biggr)x \\ & =x^{5}-x^{7}>0. \end{aligned}$$

Considering the fact $N(0)=0$, the inequality (2.18) holds. □

### Lemma 2.4

*The function* $f(x) = \lambda S(x) + (1 - \lambda)T(x) - H(x) $ *is strictly decreasing on* $(0.76,1)$, *where* $\lambda= 1 - \pi/[4\log(1 + \sqrt{2} )] = 0.1089 \ldots$ *and* $H(x)$, $S(x)$ *and* $T(x)$ *are defined as in Lemmas* 2.1, 2.2 *and* 2.3, *respectively*.

### Proof

Direct computation leads to

$$\begin{aligned}& S'(x) = 2\frac{x-\tanh^{-1}x}{(1-x^{2})^{2}(\tanh^{-1}x)^{3}}, \\& S''(x) =2\frac{\varphi(x)}{(1-x^{2})^{3}(\tanh^{-1}x)^{4}}, \end{aligned}$$

where $\varphi(x) = 3(1 + {x^{2}}){\tanh^{ - 1}}x - 3x - 4x{({\tanh^{ - 1}}x)^{2}} $. It follows that

$$ \varphi'(x) = \frac{R(x)}{1-x^{2}}, $$

where $R(x) = - 4(1 - {x^{2}}){({\tanh^{ - 1}}x)^{2}} - (6{x^{3}} + 2x) {\tanh^{ - 1}}x + 6{x^{2}} $. From (2.3), we can get

$$\begin{aligned} R(x)&< -4\bigl(1-x^{2}\bigr) \biggl(x+\frac{x^{3}}{3} \biggr)^{2}-\bigl(6x^{3}+2x\bigr) \biggl(x+ \frac{x^{3}}{3}\biggr)+6x^{2} \\ &=\frac{4}{9}x^{8}+\frac{2}{9}x^{6}- \frac{16}{3}x^{4}< 0. \end{aligned}$$

Thus $\varphi(x)$ is strictly decreasing on $(0.76,1)$. Considering the fact $\varphi(0.76) = - 0.5821\ldots<0 $, we have $\varphi(x)<0 $ for any $x \in(0.76,1) $. In other words, $S'(x) $ is strictly decreasing on $(0.76,1) $.

Let $\phi(x) = \lambda S(x) + (1 - \lambda)T(x) $. It was proved that $T'(x) $ is strictly decreasing on $(0.7,1) $ in Lemma 5 of [26]. Thus, from the monotonicity of $S'(x) $ and $T'(x) $, we have

$$ \phi'(x) < \lambda S'(0.76) + (1 - \lambda)T'(0.76) = - 0.0043 \ldots< 0 $$

for any $x \in(0.76,1) $. That is to say, $\phi(x)$ is strictly decreasing on $(0.76,1)$. Considering the monotonicity of $H(x)$ in Lemma 2.1, the proof is completed. □

### Lemma 2.5

*We have*

$$ \frac{4-11\lambda}{28}x^{4} - \frac{27\lambda+13}{45}x^{2}+ \frac{1-4 \lambda}{3}>0 $$

*for* $x \in(0,0.76)$, *where* $\lambda= 1 - \pi/[4\log(1 + \sqrt{2} )]=0.1089\ldots$ .

### Proof

Let

$$ \eta(x) = \frac{4-11\lambda}{28}x^{4} - \frac{27\lambda+13}{45}x ^{2}+\frac{1-4\lambda}{3}. $$

Then it is easy to verify that $\eta(x)$ is decreasing on $(0,\mu)$, where

$$ \mu=\sqrt{\frac{14}{15}} \times\sqrt{\frac{160\log(1+ \sqrt{2})-27\pi}{11\pi-28\log(1+\sqrt{2})}}= 1.3303 \ldots. $$

Considering $\eta(0.76) = 0.01693 \ldots>0 $, we have $\eta(x)>0$ for $x\in(0,0.76)$. □

## Main results

### Theorem 3.1

*The double inequality*

$$ \alpha L(a,b) + (1 - \alpha)T(a,b) < \mathit {NS}(a,b) < \beta L(a,b) + (1 - \beta)T(a,b) $$

*holds for any* $a,b>0$ *with* $a \ne b $ *if and only if* $\alpha\ge1/4 $ *and*

$$ \beta\le1 - \frac{\pi}{4\log(1+\sqrt{2})}= 0.1089 \ldots. $$

### Proof

Because $\mathit {NS}(a,b)$, $L(a,b)$, $T(a,b)$ are symmetric and homogeneous of degree 1, without loss of generality, we can assume that $a>b$ and $x:=(a-b)/(a+b)\in(0,1)$. Let $p\in(0,1)$ and $\lambda = 1 - \pi/[4\log(1 + \sqrt{2} )] = 0.1089 \ldots$ . Then by (1.1)-(1.3), direct computation leads to

$$\begin{aligned}& \frac{\mathit {NS}(a,b)}{A(a,b)}=\frac{x}{\sinh^{-1}x}, \\& \frac{L(a,b)}{A(a,b)}=\frac{x}{\tanh^{-1}x}, \\& \frac{T(a,b)}{A(a,b)}=\frac{x}{\tan^{-1}x}. \end{aligned}$$

Let

3.1 $$\begin{aligned} F_{t}(x) &= \frac{tL(a,b) + (1 - t)T(a,b) - M(a,b)}{A(a,b)} \\ & =t\frac{x}{\tanh^{-1}x}+(1-t)\frac{x}{\tan^{-1}x}-\frac{x}{ \sinh^{-1}x}. \end{aligned}$$

Then it follows that

3.2 $$\begin{aligned}& F_{\frac{1}{4}}\bigl(0^{+}\bigr)=0, \end{aligned}$$

3.3 $$\begin{aligned}& F_{\lambda}\bigl(0^{+}\bigr)=F_{\lambda} \bigl(1^{-}\bigr)=0. \end{aligned}$$

Differentiating $F_{t}(x) $, we have

$$\begin{aligned} F'_{t}(x)&=t\biggl[\frac{1}{\tanh^{-1}x}- \frac{x}{1-x^{2}}\frac{1}{(\tanh ^{-1}x)^{2}}\biggr] \\ &\quad{} +(1-t)\biggl[\frac{1}{\tan^{-1}x}-\frac{x}{1+x^{2}} \frac{1}{(\tan^{-1}x)^{2}}\biggr] \\ &\quad{} -\biggl[\frac{1}{\sinh^{-1}x}-\frac{x}{\sqrt{1+x^{2}}} \frac{1}{(\sinh^{-1}x)^{2}}\biggr] \\ &:=tS(x)+(1-t)T(x)-H(x), \end{aligned} $$

where $H(x)$, $S(x)$ and $T(x)$ are defined as in Lemmas 2.1-2.3, respectively.

On one hand, from inequalities (2.4), (2.5) and (2.16), we clearly see that

$$\begin{aligned} F'_{\frac{1}{4}}(x)&=\frac{1}{4}S(x)+\frac{3}{4}T(x)-H(x) \\ &< \frac{1}{4}\biggl(-\frac{2}{3}x- \frac{1}{3}x^{3}-\frac{1}{3}x^{5}\biggr)+ \frac{3}{4}\biggl(\frac{2}{3}x-\frac{1}{3}x^{3}+ \frac{2}{7}x^{5}\biggr) -\biggl( \frac{x}{3}- \frac{17}{90}x^{3}\biggr) \\ & =-\frac{13}{90}x^{3}+\frac{11}{84}x^{5}< 0 \end{aligned} $$

for any $x\in(0,1)$. It leads to

3.4 $$\begin{aligned}& F_{\frac{1}{4}}(x)< F_{\frac{1}{4}}(0)=0 \end{aligned}$$

for any $x\in(0,1)$. Thus, from (3.1) it follows that

$$\begin{aligned}& \mathit {NS}(a,b)>\frac{1}{4}L(a,b)+\frac{3}{4}T(a,b) \end{aligned}$$

for all $a,b>0$ with $a \ne b $. Considering $L(a,b)<\mathit {NS}(a,b)<T(a,b)$, we can get

3.5 $$\begin{aligned}& \mathit {NS}(a,b)>\alpha L(a,b)+(1-\alpha)T(a,b) \end{aligned}$$

for all $\alpha\geq1/4$ and $a,b>0$ with $a \ne b $.

On the other hand, from inequalities (2.3), (2.6) and (2.17), we have

$$\begin{aligned} F'_{\lambda}(x)&>-\lambda\biggl(\frac{2}{3}x+x^{3}+ \frac{x^{5}}{4}\biggr)+(1- \lambda) \biggl(\frac{2}{3}x- \frac{2}{5}x^{3}+\frac{x^{5}}{7}\biggr)-\biggl( \frac{x}{3}-\frac{x^{3}}{9}\biggr) \\ &=x\biggl[\frac{4-11\lambda}{28}x^{4}-\frac{27\lambda +13}{45}x^{2}+ \frac{1-4 \lambda}{3}\biggr] \end{aligned}$$

for $x\in(0,0.76)$. According to Lemma 2.5, we have

3.6 $$\begin{aligned}& F'_{\lambda}(x)>0 \end{aligned}$$

for $x\in(0,0.76)$. Lemma 2.4 shows that $F'_{\lambda}(x) $ is strictly decreasing on $(0.76,1)$. This fact and $F'_{\lambda}(0.76)=0.0713 \ldots>0$ together with $F'_{\lambda}(1^{-} ) = - \infty$ imply that there exists ${x_{0}}\in(0.76,1)$ such that $F_{\lambda}(x) $ is strictly increasing on $(0,x_{0}]$ and strictly decreasing on $[x_{0},1)$. Equations (3.1) and (3.3) together with the piecewise monotonicity of $F_{\lambda}(x) $ lead to the conclusion that

$$\begin{aligned} &\mathit {NS}(a,b) < \lambda L(a,b) + (1 - \lambda)T(a,b) \end{aligned}$$

for all $a,b>0$ with $a\ne b$. Considering $L(a,b)< M(a,b)< T(a,b)$, we can get

3.7 $$\begin{aligned} &\mathit {NS}(a,b) < \beta L(a,b) + (1 - \beta)T(a,b) \end{aligned}$$

holds for $\beta\leq\lambda$ and all $a,b > 0$ with $a \ne b$.

Finally, we prove that $L(a,b)/4+3T(a,b)/4$ and $\lambda L(a,b)+(1- \lambda)T(a,b)$ are the best possible lower and upper mean bound for the Neuman-Sándor mean $M(a,b)$.

For any $\epsilon_{1}, \epsilon_{2}>0$, let $t_{1}=1/4-\epsilon_{1}$, $t_{2}=\lambda+\epsilon_{2}$. Then one can get

3.8 $$\begin{aligned}& F_{t_{1}}(x)=\biggl(\frac{1}{4}-\epsilon_{1}\biggr) \frac{x}{\tanh^{-1}x}+\biggl( \frac{3}{4}+\epsilon_{1}\biggr) \frac{x}{\tan^{-1}x}-\frac{x}{\sinh^{-1}x}, \end{aligned}$$

3.9 $$\begin{aligned}& F_{t_{2}}(x)=(\lambda+\epsilon_{2})\frac{x}{\tanh^{-1}x}+(1- \lambda -\epsilon_{2})\frac{x}{\tan^{-1}x}-\frac{x}{\sinh^{-1}x}. \end{aligned}$$

Let $x_{1}\rightarrow0^{+}$ and $x_{2}\rightarrow1^{-}$, then the Taylor expansion leads to

3.10 $$\begin{aligned}& F_{t_{1}}(x_{1})=\frac{2}{3}\epsilon_{1} x_{1}^{2}+O\bigl(x_{1}^{4}\bigr), \end{aligned}$$

3.11 $$\begin{aligned}& F_{t_{2}}(x_{2})=-4\epsilon_{2}/ \pi+O(x_{2}-1). \end{aligned}$$

Equations (3.8) and (3.10) imply that if $\alpha<1/4$, then, for any $\epsilon_{1}>0$, there exists ${\sigma_{1}} \in(0,1)$ such that $\mathit {NS}(a,b) < (1/4-\epsilon_{1})L(a,b) + (3/4 - \epsilon_{1})T(a,b) $ for all *a*, *b* with $(a - b)/(a + b) \in(0,{\sigma_{1}}) $.

Equations (3.9) and (3.11) imply that if $\beta>\lambda$, then, for any $\epsilon_{2}>0$, there exists ${\sigma_{2}} \in(0,1)$ such that $\mathit {NS}(a,b) > (\lambda+\epsilon_{2})L(a,b) + (1 - \lambda- \epsilon_{2})T(a,b) $ for all *a*, *b* with $(a - b)/(a + b) \in(1 - {\sigma_{2}},1) $. □

## Conclusion

In the article, we give the sharp upper and lower bounds for Neuman-Sándor mean in terms of the linear convex combination of the logarithmic and second Seiffert means.
